# The House-Fly and Public Health

**Published:** 1913-06-21

**Authors:** 


### THE HOUSE-FLY AND PUBLIC HEALTH.

The importance of the common house-fly in the realm of public health is only just beginning to be realised. The active rôle of this insect in the propagation of disease, especially amongst children, is now not a matter of hypothesis but of actual fact. Yet the public, and even the medical profession, have been slow to attribute to the house-fly all those crimes which the sanitarians and bacteriologists have been busily engaged in laying to its charge. The great achievements which recent years have seen following the practical application of the knowledge gained as to the mode of transmission of malarial fevers have been accepted with gratitude, and the whole world has benefited enormously: witness the successful reduction of mosquitos in Panama and Port Said and the almost complete abolition of certain scourges from these districts. But the part played by the domestic fly has not yet been considered seriously. Organised attempts at reduction of these insects have not yet come into the sphere of public health politics. Perhaps this is because the proofs of the damage caused by flies are not so thoroughly established as those in connection with mosquitos. Moreover, flies do not bite and we are thoroughly accustomed to them. There is more scientific glamour attached to foreign mosquitos and yellow fever than to the house-fly and its connection with infantile mortality rates.

Signs are not wanting, however, to show that at last public opinion is being awakened to the dangers which lurk in and upon the body of this insect. Fly campaigns have, indeed, been organised, and even prizes awarded for "catches" of dead flies. But this method is not the rational one. It attacks the problem at the wrong end, and fails to touch the root of the evil. The pest must be destroyed, not in the final or *imago* stage, but in the earliest or egg stage, or at least in the larval or maggot stage. This point has been strongly insisted upon in a recent book, "The Reduction of Domestic Flies," by E. H. Ross (Murray, 5s.). Flies in their larval stage can easily be killed in their thousands, whereas in their complete insect form they can only be dealt with at best in hundreds. The house-fly breeds in all sorts of filth, but stable manure is the commonest lair. The female fly finds this a very suitable place in which to lay her eggs, and in horse-dung the maggot or grub lives for about five days or more, according to the temperature, and then becomes a chrysalis (pupa). The next stage may last another five days or so, after which the fully developed flying insect or imago emerges to do deadly work amongst our infants and children. Although several diseases may be transmitted by flies besides typhoid fever—for instance, ophthalmia—there is one most important disease which is to a very large extent carried by flies—namely, infantile diarrhœa or zymotic enteritis. This complaint, it need hardly be mentioned, carries off every year thousands of young children in London alone.

The way to lessen this mortality is to attack the breeding places of the fly; far more lives would be saved by an energetic campaign in this direction than by all the sea-water polyclinics in existence and to come. An anti-fly campaign would fall naturally under the ægis of the local sanitary authority and be organised by the medical officer of health. Without going into details, it may be indicated that a systematic inspection of and report on the stables in all the districts of the town is one of the first steps; this is to be followed by a continuous orderly attack on the breeding places and the removal of manure and rubbish heaps at least once a week. As the fully developed flying insect takes about ten days to mature, a weekly "clean up" is sufficient for practical purposes. Now that the hot weather is impressing the fly nuisance on the public mind, the chance of inaugurating a campaign against the fly danger must not be wasted. Such a campaign could not fail to produce a very real effect on the mortality bills.

